# Risk of acute atherosclerotic cardiovascular disease in patients with acute and chronic pancreatitis

**DOI:** 10.1038/s41598-021-99915-4

**Published:** 2021-10-22

**Authors:** Li-Chin Sung, Chuen-Chau Chang, Chao-Shun Lin, Chun-Chieh Yeh, Yih-Giun Cherng, Ta-Liang Chen, Chien-Chang Liao

**Affiliations:** 1 Division of Cardiology, Department of Internal Medicine, School of Medicine, College of Medicine, Taipei Medical University, Taipei, Taiwan; 2 Division of Cardiology, Department of Internal Medicine, Shuang Ho Hospital, Taipei Medical University, New Taipei City, Taiwan; 3 Taipei Heart Institute, Taipei Medical University, Taipei, Taiwan; 4 Department of Anesthesiology, School of Medicine, College of Medicine, Taipei Medical University, Taipei, Taiwan; 5 Department of Anesthesiology, Taipei Medical University Hospital, 252 Wu-Xing Street, Taipei, Taiwan; 6 Anesthesiology and Health Policy Research Center, Taipei Medical University Hospital, Taipei, Taiwan; 7 Department of Surgery, China Medical University Hospital, Taichung, Taiwan; 8 Department of Surgery, University of Illinois, Chicago, IL USA; 9 Department of Anesthesiology, Shuang Ho Hospital, Taipei Medical University, Taipei, Taiwan; 10 Department of Anesthesiology, Wang Fang Hospital, Taipei Medical University, Taipei, Taiwan; 11 Research Center of Big Data and Meta-Analysis, Wan Fang Hospital, Taipei Medical University, Taipei, Taiwan; 12 School of Chinese Medicine, College of Chinese Medicine, China Medical University, Taichung, Taiwan

**Keywords:** Cardiology, Diseases, Gastroenterology, Health care, Risk factors

## Abstract

The association between pancreatitis and acute myocardial infarction or stroke remains incompletely understood. This study aimed to evaluate the long-term risk of acute atherosclerotic cardiovascular disease (ASCVD) in people with acute and chronic pancreatitis. Using research database of Taiwan's National Health Insurance, we identified 2678 patients aged ≥ 20 years with newly diagnosed pancreatitis in 2000–2008. A cohort of 10,825 adults without pancreatitis was selected for comparison, with matching by age and sex. Both cohorts were followed from 2000 to the end of 2013, and incident acute ASCVD was identified during the follow-up period. Adjusted hazard ratios (HRs) and 95% confidence intervals (CIs) of acute ASCVD associated with pancreatitis were calculated. Compared with the comparison cohort, the adjusted HR of acute ASCVD were 1.76 (95% CI 1.47–2.12) and 3.42 (95% CI 1.69–6.94) for people with acute pancreatitis and chronic pancreatitis, respectively. A history of alcohol-related illness (HR 9.49, 95% CI 3.78–23.8), liver cirrhosis (HR 7.31, 95% CI 1.81–29.5), and diabetes (HR 6.89, 95% CI 2.18–21.8) may worsen the risk of acute ASCVD in patients with chronic pancreatitis. Compared with people had no pancreatitis, patients with acute pancreatitis who had alcohol-related illness (HR 4.66, 95% CI 3.24–6.70), liver cirrhosis (HR 4.44, 95% CI 3.05–6.47), and diabetes (HR 2.61, 95% CI 2.03–3.36) were at increased risk of acute ASCVD. However, the cumulative use of metformin was associated with a reduced risk of acute ASCVD in the acute pancreatitis cohort (HR 0.30, 95% CI 0.17–0.50). Compared with the control group, patients with acute or chronic pancreatitis were more likely to have an increased risk of acute ASCVD, while the use of metformin reduced the risk of acute ASCVD. Our findings warrant a survey and education on acute ASCVD for patients with acute and chronic pancreatitis.

## Introduction

Coronary artery disease and stroke are the second and third leading causes of death in the world, respectively^1^. Atherosclerosis is a common pathological process characterized by the accumulation of lipids and lymphocytes/macrophages in the walls of large and medium-sized arteries^2,3^. Atherosclerotic cardiovascular disease (ASCVD), including coronary artery disease and ischemic stroke, accounts for the majority of cardiovascular mortality, imposes enormous economic burdens and continues to be a major public health issue worldwide^1,4^.

Pancreatitis, which is characterized by inflammation of the pancreas, have been classified in either acute, recurrent acute or chronic forms^5,6^. Acute pancreatitis often leads to a number of sequelae long after clinical resolution, and should no longer be considered a self-limited disease^6^. Around 21% of patients suffering a first acute pancreatitis will develop recurrent acute pancreatitis. Of those developing recurrent acute pancreatitis, up to 36% will develop chronic pancreatitis^5^. Alcohol abuse and gallstones are common etiologies for acute pancreatitis, accounting for over 80% of all cases^5^. In most areas, the gallstone is the most common etiology following by idiopathic etiology. Acute pancreatitis is associated with profound hypotension, hyperglycemia and increased circulating levels of inflammatory cytokines/proteolytic enzymes^7,8^. Chronic pancreatitis is a chronic inflammatory and fibrotic disease of the pancreas with a prevalence of 42 to 73 per 100,000 adults in the United States^9^. Chronic pancreatitis often results in chronic abdominal pain and is most commonly caused by excessive alcohol use, smoking, or genetic mutations^9^. Suggestions of treatment included alcohol and smoking cessation, pain control, replacement of pancreatic insufficiency, or mechanical drainage of obstructed pancreatic ducts for some patients^5,9–11^.

Some studies have suggested that patients with acute or chronic pancreatitis are more likely to have acute ASCVD, including acute myocardial infarction and stroke, compared with those without pancreatitis^7,8,10,11^. However, there is heterogeneity in these previous reports because of study limitations, such as small sample sizes^8^, inadequate control of confounding factors^7,8,10,11^, and unclear definitions of pancreatitis cases^7,9,11^. In addition, the influence of pancreatitis severity on the risk of acute ASCVD is unclear. Using research data from the health insurance program in Taiwan, we conducted this retrospective cohort study to evaluate the risk of acute ASCVD in patients with acute and chronic pancreatitis.

## Methods

### Source of data

In this study, we used research data from the health insurance program, and it covers at least 99% of residents in Taiwan. The research database included the patient’s basic characteristics, examination, diagnoses, prescriptions, and treatments of inpatient and outpatient medical services. The detailed description and reviews of this database were reported previously^7,8,10,11^. Our study waived the need of informed consent and it was approved by the institutional review board of Taipei Medical University (TMU-JIRB-201701050; TMU-JIRB-201705063; TMU-JIRB-201705065; TMU-JIRB-201808012; TMU-JIRB-201912046). All methods were carried out in accordance with relevant guidelines and regulations.

### Study design

We conducted this retrospective cohort study using a random sample of 1 million beneficiaries from the research database and there is no direct participation of patients in the current study. The database of Taiwan’s National Health Insurance was available since 1996. We identified 2737 patients aged over 20 years who were newly diagnosed with pancreatitis in the period 2000–2008; 10,948 persons without a history of pancreatitis were selected as the control group, with frequency matching by age and sex (case–control ratio nearly 1:4). In this study, the wash-out period was 4 years (confirming both cohorts had no physician’s diagnosis of pancreatitis within past 4 years). To strictly identify pancreatitis cases, we defined pancreatitis by requiring inpatient care with pancreatitis as the physician's primary diagnosis. The pancreatitis and non-pancreatitis cohorts had no history of acute ASCVD at the start, and both were followed up from the index date until December 31, 2013, or until they were censored because of death. The event of acute ASCVD was considered the outcome during the follow-up period.

### Measures and definitions

The low-income patients were identified according to the criteria from the Ministry of Health and Welfare. For indentifying medical conditions and major outcomes, we used the physician’s diagnoses and the International Classification of Diseases-Clinical Modification, 9th edition (ICD-9-CM) including acute pancreatitis (code 577.0), chronic pancreatitis (code 577.1), stroke (codes 430–437), acute myocardial infarction (codes 410), diabetes (codes 250), hypertension (codes 401–405), mental disorders (codes 290–319), alcohol-related illness (codes 291, 303, 305, 571.0, 571.1, 571.2, 571.3), liver cirrhosis (codes 571.2, 571.5, 571.6), hyperlipidemia (codes 272.0, 272.1, and 272.2), chronic obstructive pulmonary disease (codes 491, 492, 496), and cholecystitis (codes 574). The insurance codes (D8, D9) were used to identify medical dialysis of kidney.

Based on our previous drug-related studies^12–15^, we defined people who visited medical care (included outpatient, inpatient, and emergency care) and received a physician’s prescription for metformin under the coverage of Taiwan’s National Health Insurance Program. The prescription of metformin was identified from the medication records of payment of Taiwan’s National Health Insurance Program. In this study, we check the date of prescription of metformin and confirm that the use of metformin fall at time point after an attack of pancreatitis. We collected and calculated the cumulative use of metformin during the follow-up period.

To avoid immortal time bias (which might result in overestimation of the intervention’s beneficial effects) in the metformin group, we followed up the pancreatitis patients from the first date when they received metformin after index the diagnosis of pancreatitis until December 31, 2013 or until an event (developed a acute ASCVD, lost to follow-up, or death). The follow-up time, in person-years, was calculated for each pancreatitis patient from the index date to the end-point. We compared the risk of an incident acute ASCVD during the follow-up period between the pancreatitis patients who received metformin treatment and those who did not.

### Statistical analyses

We compared sociodemographic factors (age, sex and low income) and coexisting medical conditions (diabetes, hypertension, mental disorders, alcohol-related illness, liver cirrhosis, hyperlipidemia, chronic obstructive pulmonary disease, cholecystitis, and renal dialysis) in the cohorts of patients with and without pancreatitis using chi-square tests. In the overall population and subgroup analysis stratified by age and sex, we used multivariate Cox proportional hazard models to calculate the adjusted hazard ratios (HRs) and 95% confidence intervals (CIs) for acute ASCVD associated with pancreatitis. The effects of pancreatitis-related diseases and metformin medication on the risk of acute ASCVD in patients with pancreatitis were also evaluated using Cox proportional hazard models.

## Results

Under the frequency matching procedure, there were no significant differences in age or sex between subjects with and without pancreatitis (Table 1). Higher proportions of low income, diabetes, hypertension, mental disorders, alcohol-related illness, liver cirrhosis, hyperlipidemia, chronic obstructive pulmonary disease, cholecystitis, and renal dialysis were found in pancreatitis patients than in subjects without pancreatitis (all p < 0.05).

**Table 1:** Baseline characteristics of people with and without pancreatitis.

	No pancreatitis (n = 10,825)		Pancreatitis (n = 2678)		p-value
	n	(%)	n	n	(%)
**Sex**					
Female	3284	(30.3)	808	3284	(30.3)
Male	7541	(69.7)	1870	7541	(69.7)
**Age, years**					
20–29	808	(7.5)	201	808	(7.5)
30–39	2311	(21.4)	575	2311	(21.4)
40–49	2585	(23.9)	641	2585	(23.9)
50–59	1661	(15.3)	411	1661	(15.3)
60–69	1493	(13.8)	365	1493	(13.8)
70–79	1341	(12.4)	336	1341	(12.4)
≥ 80	626	(5.8)	149	626	(5.8)
**Low income**					
No	10,488	(96.9)	2486	10,488	(96.9)
Yes	337	(3.1)	192	337	(3.1)
**Medical conditions**					
Diabetes	986	(9.1)	600	986	(9.1)
Hypertension	1937	(17.9)	538	1937	(17.9)
Mental disorders	1034	(9.6)	401	1034	(9.6)
Alcohol-related illness	91	(0.8)	294	91	(0.8)
Liver cirrhosis	175	(1.6)	181	175	(1.6)
Hyperlipidemia	419	(3.9)	156	419	(3.9)
COPD	351	(3.2)	121	351	(3.2)
Cholecystitis	121	(1.1)	120	121	(1.1)
Renal dialysis	61	(0.6)	51	61	(0.6)

*COPD* chronic obstructive pulmonary disease.

As shown in Table 2, a higher incidence of acute ASCVD was found in patients with acute (11.8 per 1000 person-years) and chronic (17.7 per 1000 person-years) pancreatitis than in those without pancreatitis (6.81 per 1000 person-years, p < 0.0001) during the follow-up period. The corresponding HR of acute ASCVD associated with acute pancreatitis and chronic pancreatitis were 1.76 (95% CI 1.47–2.12) and 3.42 (95% CI 1.69–6.94), respectively. Among people with 1 (HR 2.01, 95% CI 1.49–2.71) and ≥ 2 (HR 2.02, 95% CI 1.53–2.67) medical conditions, pancreatitis was associated with the risk of acute ASCVD. Table S1 showed the stratified analyses (by sex and age) for the risk of acute ASCVD in patients with acute and chronic pancreatitis.

**Table 2:** The long-term risk of acute ASCVD for people with and without pancreatitis.

	Long-term risk of acute ASCVD					
	n	Events	Person-years	Incidence^b^	HR	95% CI^a^
No pancreatitis	10,825	647	94,957	6.81	1.00	Reference
Pancreatitis	2678	267	22,335	12.0	1.80	1.50–2.15
Acute pancreatitis	2607	257	21,769	11.8	1.76	1.47–2.12
Chronic pancreatitis	71	10	565	17.7	3.42	1.69–6.94
**0 medical condition**						
No pancreatitis	7196	195	63,237	3.08	1.00	Reference
Pancreatitis	1330	46	10,880	4.23	1.22	0.83–1.81
Acute pancreatitis	1295	44	10,597	4.15	1.17	0.78–1.76
Chronic pancreatitis	35	2	283	7.07	3.14	0.78–12.7
**1 medical condition**						
No pancreatitis	2411	231	21,397	10.8	1.00	Reference
Pancreatitis	764	91	6757	13.5	2.01	1.49–2.71
Acute pancreatitis	747	88	6604	13.3	2.00	1.48–2.70
Chronic pancreatitis	17	3	153	19.6	2.70	0.66–11.0
**≥ 2 medical conditions**						
No pancreatitis	1218	221	10,322	21.4	1.00	Reference
Pancreatitis	584	130	4698	27.7	2.02	1.53–2.67
Acute pancreatitis	565	125	4569	27.4	1.98	1.50–2.62
Chronic pancreatitis	19	5	129	38.8	4.06	1.48–11.1

*ASCVD* atherosclerotic cardiovascular disease, *CI* confidence interval, *HR* hazard ratio.

^a^Adjusted for all covariates listed in Table 1.

^b^Per 1000 person-years.

Compared with people without pancreatitis (Table 3), those with acute pancreatitis who had alcohol-related illness (HR 4.66, 95% CI 3.24–6.70), liver cirrhosis (HR 4.44, 95% CI 3.05–6.47), diabetes (HR 2.61, 95% CI 2.03–3.36), and cholecystitis (HR 2.24, 95% CI 1.30–3.87) were more likely to have acute ASCVD. The increased risk of ASCVD was found in patients with chronic pancreatitis who had alcohol-related illness (HR 9.49, 95% CI 3.78–23.8), liver cirrhosis (HR 7.31, 95% CI 1.81–29.5), and diabetes (HR 6.89, 95% CI 2.18–21.8). In patients with acute pancreatitis, the use of metformin ≥ 2 times (HR 0.30, 95% CI 0.17–0.50) was associated with a reduced risk of ASCVD.

**Table 3:** The effects of medical conditions on long-term risk of acute ASCVD in patients with pancreatitis.

	Long-term risk of acute ASCVD				
	n	Events	Incidence^b^	HR	95% CI
People without pancreatitis	10,825	647	6.81	1.00	Reference
**Pancreatitis patients had ARI**	294	53	22.7	4.89	3.44–6.96
Acute pancreatitis patients had ARI	279	48	21.4	4.66	3.24–6.70
Chronic pancreatitis patients had ARI	15	5	50.5	9.49	3.78–23.8
**Pancreatitis patients had liver cirrhosis**	181	35	22.9	4.53	3.14–6.54
Acute pancreatitis patients had liver cirrhosis	171	33	22.8	4.44	3.05–6.47
Chronic pancreatitis patients had liver cirrhosis	10	2	25.6	7.31	1.81–29.5
**Pancreatitis patients had diabetes**	600	97	18.9	2.67	2.08–3.42
Acute pancreatitis patients had diabetes	586	93	18.4	2.61	2.03–3.36
Chronic pancreatitis patients had diabetes	14	4	40.8	6.89	2.18–21.8
**Pancreatitis patients had cholecystitis**	120	31	34.5	2.18	1.26–3.76
Acute pancreatitis patients had cholecystitis	118	30	33.9	2.24	1.30–3.87
Chronic pancreatitis patients had cholecystitis	2	1	78.4	–	–
**People had pancreatitis**					
Without use of metformin	2293	242	12.8	1.00	Reference
Use 1 time of metformin	43	4	9.83	0.45	0.16–1.23
Use ≥ 2 times of metformin	342	21	6.80	0.28	0.16–0.47
**People had acute pancreatitis**					
Without use of metformin	2228	232	12.7	1.00	Reference
Use 1 time of metformin	43	4	9.83	0.46	0.17–1.26
Use ≥ 2 times of metformin	336	21	6.91	0.30	0.17–0.50
**People had chronic pancreatitis**					
Without use of metformin	65	10	19.5	1.00	Reference
Use 1 time of metformin	0	0	0.0	–	–
Use ≥ 2 times of metformin	6	0	0.0	–	–

*ARI* alcohol-related illness, *ASCVD* atherosclerotic cardiovascular disease, *CI* confidence interval, *HR* hazard ratio.

^a^Adjusted for all covariates listed in Table 1.

^b^Per 1000 person-years.

During the follow-up period (Table 4), patients with acute pancreatitis had an increased risk of acute myocardial infarction (HR 1.75, 95% CI 1.11–2.74) or stroke (HR 1.76, 95% CI 1.44–2.15). The risks of hemorrhagic stroke (HR 2.14, 95% CI 1.42–3.23), ischemic stroke (HR 1.46, 95% CI 1.05–2.02), and other stroke (HR 1.62, 95% CI 1.17–2.23) were associated with acute pancreatitis. Chronic pancreatitis was associated with risk of stroke (HR 4.20, 95% CI 2.07–8.56), included ischemic stroke (HR 6.20, 95% CI 2.25–17.1) and other stroke (HR 3.78, 95% CI 1.19–12.0).

**Table 4:** Long-term risk of acute myocardial infarction and stroke in patient with pancreatitis.

	Long-term risk of acute ASCVD					
	n	Events	Person-years	Incidence^b^	HR	95% CI^a^
**Risk of AMI**						
No pancreatitis	10,825	98	97,590	1.00	1.00	Reference
Pancreatitis	2678	50	23,325	2.14	1.71	1.09–2.68
Acute pancreatitis	2607	50	22,711	2.20	1.75	1.11–2.74
Chronic pancreatitis	71	0	614	0	–	–
**Risk of all stroke**						
No pancreatitis	10,825	549	95,411	5.75	1.00	Reference
Pancreatitis	2678	217	22,505	9.64	1.81	1.49–2.21
Acute pancreatitis	2607	207	21,939	9.44	1.76	1.44–2.15
Chronic pancreatitis	71	10	565	17.7	4.20	2.07–8.56
**Risk of hemorrhagic stroke**						
No pancreatitis	10,825	90	97,668	0.92	1.00	Reference
Pancreatitis	2678	50	23,284	2.15	2.12	1.41–3.19
Acute pancreatitis	2607	49	22,678	2.16	2.14	1.42–3.23
Chronic pancreatitis	71	1	606	1.65	1.49	0.20–10.9
**Risk of ischemic stroke**						
No pancreatitis	10,825	239	96,872	2.47	1.00	Reference
Pancreatitis	2678	78	23,143	3.37	1.54	1.12–2.12
Acute pancreatitis	2607	73	22,550	3.24	1.46	1.05–2.02
Chronic pancreatitis	71	5	594	8.42	6.20	2.25–17.1
**Risk of other stroke**						
No pancreatitis	10,825	220	96,960	2.27	1.00	Reference
Pancreatitis	2678	89	23,067	3.86	1.67	1.21–2.29
Acute pancreatitis	2607	85	22,473	3.78	1.62	1.17–2.23
Chronic pancreatitis	71	4	595	6.72	3.78	1.19–12.0

*ASCVD* atherosclerotic cardiovascular disease, *AMI* acute myocardial infarction, *CI* confidence interval, *HR* hazard ratio.

^a^Adjusted for all covariates listed in Table 1.

^b^Per 1000 person-years.

Table S2 showed the stratified analyses for the risk of acute myocardial infarction in patients with acute and chronic pancreatitis. Table S3 showed the stratified analyses for the risk of stroke in patients with acute and chronic pancreatitis.

## Discussion

Our population-based cohort study showed that patients with pancreatitis had increased risks of acute ASCVD, including acute myocardial infarction and stroke. The association between pancreatitis and acute ASCVD was significant in various subgroups. Patients with pancreatitis showed higher risks of acute ASCVD as well as complications such as liver cirrhosis, diabetes, and cholecystitis. Our study also reported the safety of metformin in patients with pancreatitis.

The association between pancreatitis and acute ASCVD could not be clarified completely in this observational study, we reviewed some relevant studies to explain the mechanism. First, the endothelium is considered a regulator of vascular homeostasis. Inflammation may cause endothelial dysfunction, which contributes to the initiation and progression of atherosclerosis^2^. Inflammation of the pancreas causes the aggregation of neutrophils, macrophages, and injured pancreatic acinar cells, releasing cytokines such as interleukin (IL)-10, IL-6, tumor necrosis factor-α, transforming growth factor-β, platelet-derived growth factor, and the chemokine monocyte chemoattractant protein-1^11,16^. These inflammatory cytokines play a pivotal role in the pathogenesis of atherosclerosis^17,18^. These inflammatory cells further enhance inflammation and tend to precipitate unstable or ruptured atherosclerotic plaques. Second, severe hemodynamic disturbances such as profound hypotension and hypovolemia decrease coronary artery or cerebrovascular perfusion^8^. Third, metabolic disturbances include hyperglycemia and hyperlipidemia, which exert equal stimulatory effects on platelet activation and vascular events^19,20^.

The long-term effect of gallstone disease on cardiovascular outcomes is highly debated^21–24^. Although we investigated that pancreatitis patients with cholecystitis had increased risk of acute ASCVD in this study, we recommend further prospective studies to examine this epiphenomenon.

Post-pancreatitis diabetes mellitus is an exemplar secondary diabetes that represents a sequela of pancreatitis—the most common disease of the exocrine pancreas^25,26^. People have at least two-fold higher lifetime risk of developing diabetes after an attack of pancreatitis than those in the general population^25^. Both basic research and population-based study provide strong evidence suggesting that hypertriglyceridemia could be involved in the pathophysiology of acute ASCVD^20,27,28^. We considered that the effect of hypertriglyceridemia as the etiology of acute pancreatitis as well as a risk factors for severe disease and the potential link with cardiovascular disorders.

It has been shown that the use of metformin is beneficial for reducing the risk of cardiovascular events in diabetic patients^24,29^. Growing evidence suggests that metformin has a protective effect against acute ASCVD beyond its hypoglycemic effects^29,30^. Possible explanations for the cardio-protective role of metformin may be due to its pleiotropic effects, including antioxidant, improved endothelial cell function, decreased smooth muscle cell migration, decreased cholesterol deposition and anti‑inflammatory activities, which may reduce atherosclerotic plaques^29,31^. Furthermore, metformin has been reported to reduce the levels of certain inflammatory cytokines involved in pancreatitis, as mentioned previously^30^. Our study is the first to report that the use of metformin is associated with a reduced risk of acute ASCVD in patients with pancreatitis. However, these interesting findings need to be verified by future randomized clinical trials.

Among people with post-pancreatitis diabetes mellitus, ever users of metformin had a significantly lower mortality, whereas ever-users of insulin did not have a significantly changed risk of mortality compared with never users of antidiabetic medications^32^. Therefore, metformin promotes a survival benefit in people with post-pancreatitis diabetes mellitus. Previous studies found that 23% and 30% of people after the first attack of acute pancreatitis and chronic pancreatitis developed post-pancreatitis diabetes mellitus, respectively^25^. Individuals with post-pancreatitis diabetes mellitus had higher rate of all-cause mortality than those with type 2 diabetes and metformin monotherapy is recommended as the first-line therapy for post-pancreatitis diabetes mellitus^25^.

Emerging data suggest that the acute, recurrent acute and chronic forms of pancreatitis represent a disease continuum^33^. Although clinical factors and pancreatic enzymes have been identified, discriminating between acute and chronic pancreatitis in individual patients remains difficult. We included patients with pancreatitis irrespective of chronicity to ensure diagnostic accuracy, and our report is comparable with previous studies^7,10,11,34,35^.

The limitations of this present study are as follows. First, information on each individual’s socioeconomic characteristics, lifestyle factors, physical examination results, laboratory measurements, and imaging results was unavailable in Taiwan’s National Health Insurance database. Smoking is an important factor for the association between pancreatitis and ASCVD. It is one of our study limitations that the information of smoking is not available in the database of Taiwan’s National Health Insurance. Second, although our results showed that use of metformin is associated with a decreased risk of acute ASCVD, the compliance of medication intake is one of study limitations.

In conclusion, we reported that patients with acute or chronic pancreatitis were more likely to have acute ASCVD, particularly those with pancreas-related comorbidities, while the use of metformin is associated with a reduced risk of acute ASCVD. Our results highlight the importance of using a multidisciplinary team to adopt an integrated approach to prevent patients with pancreatitis from developing acute ASCVD. Further clinical trials are warranted to provide more evidence regarding the beneficial effects of metformin on reducing acute ASCVD in this susceptible population.

## Supplementary Information

Supplementary Tables.

## Abbreviations

ICD-9-CM: International Classification of Diseases, 9th Revision, Clinical Modification
CI: Confidence interval
HR: Hazard ratio
ASCVD: Atherosclerotic cardiovascular disease
