# Supplementary material for: Risk of acute atherosclerotic cardiovascular disease in patients with acute and chronic pancreatitis
Source: Sci Rep. 2021 Oct 22;11:20907. doi: 10.1038/s41598-021-99915-4 (PMC8536656; doi:10.1038/s41598-021-99915-4)
Supplement: Supplementary file 1 — Supplementary Tables. [file 41598_2021_99915_MOESM1_ESM.docx]

| **Table S1.** The stratified analyses (by sex and age) for the risk of acute ASCVD in patients with acute and chronic pancreatitis | | | | | | |
| --- | --- | --- | --- | --- | --- | --- |
|  | Long-term risk of acute ASCVD | | | | | |
|  | N | Events | Person-years | Incidence^b^ | HR | (95% CI)^a^ |
| Female |  |  |  |  |  |  |
| No Pancreatitis | 3284 | 213 | 28341 | 7.52 | 1.00 | (reference) |
| Pancreatitis | 808 | 82 | 6668 | 12.3 | 1.93 | (1.36-2.74) |
| Acute pancreatitis | 793 | 81 | 6543 | 12.4 | 1.95 | (1.38-2.77) |
| Chronic pancreatitis | 15 | 1 | 125 | 8.00 | - | - |
| Male |  |  |  |  |  |  |
| No Pancreatitis | 7541 | 434 | 66616 | 6.51 | 1.00 | (reference) |
| Pancreatitis | 1870 | 185 | 15667 | 11.8 | 1.78 | (1.44-2.20) |
| Acute pancreatitis | 1814 | 176 | 15226 | 11.6 | 1.72 | (1.39-2.13) |
| Chronic pancreatitis | 56 | 9 | 441 | 20.4 | 4.29 | (2.10-8.74) |
| Age 20-39 years |  |  |  |  |  |  |
| No Pancreatitis | 3119 | 28 | 29174 | 0.96 | 1.00 | (reference) |
| Pancreatitis | 776 | 28 | 7025 | 3.99 | 2.69 | (1.49-4.85) |
| Acute pancreatitis | 759 | 28 | 6857 | 4.08 | 2.74 | (1.52-4.95) |
| Chronic pancreatitis | 17 | 0 | 168 | 0.00 | - | - |
| Age 40-64 years |  |  |  |  |  |  |
| No Pancreatitis | 2585 | 80 | 23701 | 3.38 | 1.00 | (reference) |
| Pancreatitis | 641 | 53 | 5541 | 9.57 | 2.12 | (1.46-3.06) |
| Acute pancreatitis | 614 | 49 | 5322 | 9.21 | 2.05 | (1.40-2.98) |
| Chronic pancreatitis | 27 | 4 | 219 | 18.3 | 3.85 | (1.36-10.9) |
| Age ≥65 years |  |  |  |  |  |  |
| No Pancreatitis | 5121 | 539 | 42082 | 12.8 | 1.00 | (reference) |
| Pancreatitis | 1261 | 186 | 9768 | 19.0 | 1.31 | (1.11-1.56) |
| Acute pancreatitis | 1234 | 180 | 9590 | 18.8 | 1.29 | (1.09-1.53) |
| Chronic pancreatitis | 27 | 6 | 178 | 33.7 | 2.66 | (1.19-5.96) |
| ASCVD, atherosclerotic cardiovascular disease; CI, confidence interval; HR, hazard ratio.  ^a^Adjusted for all covariates listed in Table 1.  ^b^Per 1000 person-years. | | | | | | |

| **Table S2.** The stratified analyses for the risk of acute myocardial infarction in patients with acute and chronic pancreatitis | | | | | | |
| --- | --- | --- | --- | --- | --- | --- |
|  | Long-term risk of AMI | | | | | |
|  | N | Events | Person-years | Incidence^b^ | HR | (95% CI)^a^ |
| Female |  |  |  |  |  |  |
| No Pancreatitis | 3284 | 23 | 29330 | 0.78 | 1.00 | (reference) |
| Pancreatitis | 808 | 12 | 6991 | 1.72 | 2.26 | (0.76-6.77) |
| Acute pancreatitis | 793 | 12 | 6853 | 1.75 | 2.28 | (0.76-6.83) |
| Chronic pancreatitis | 15 | 0 | 138 | 0.00 | - | - |
| Male |  |  |  |  |  |  |
| No Pancreatitis | 7541 | 75 | 68260 | 1.10 | 1.00 | (reference) |
| Pancreatitis | 1870 | 38 | 16333 | 2.33 | 1.63 | (0.99-2.69) |
| Acute pancreatitis | 1814 | 38 | 15858 | 2.40 | 1.67 | (1.02-2.76) |
| Chronic pancreatitis | 56 | 0 | 476 | 0.00 | - | - |
| Age 20-39 years |  |  |  |  |  |  |
| No Pancreatitis | 3119 | 3 | 29264 | 0.10 | 1.00 | (reference) |
| Pancreatitis | 776 | 4 | 7100 | 0.56 | 4.30 | (0.84-22.0) |
| Acute pancreatitis | 759 | 4 | 6931 | 0.58 | 4.39 | (0.85-22.5) |
| Chronic pancreatitis | 17 | 0 | 168 | 0.00 | - | - |
| Age 40-64 years |  |  |  |  |  |  |
| No Pancreatitis | 5036 | 47 | 46063 | 1.02 | 1.00 | (reference) |
| Pancreatitis | 1244 | 20 | 11058 | 1.81 | 1.30 | (0.75-2.24) |
| Acute pancreatitis | 1200 | 20 | 10703 | 1.87 | 1.33 | (0.77-2.30) |
| Chronic pancreatitis | 44 | 0 | 356 | 0.00 | - | - |
| Age ≥65 years |  |  |  |  |  |  |
| No Pancreatitis | 2670 | 48 | 22263 | 2.16 | 1.00 | (reference) |
| Pancreatitis | 658 | 26 | 5167 | 5.03 | 2.12 | (1.31-3.45) |
| Acute pancreatitis | 648 | 26 | 5077 | 5.12 | 2.16 | (1.33-3.51) |
| Chronic pancreatitis | 10 | 0 | 90 | 0.00 | - | - |
| 0 medical condition |  |  |  |  |  |  |
| No Pancreatitis | 7196 | 35 | 63923 | 0.55 | 1.00 | (reference) |
| Pancreatitis | 1330 | 14 | 11017 | 1.27 | 2.18 | (1.04-4.57) |
| Acute pancreatitis | 1295 | 14 | 10732 | 1.30 | 2.24 | (1.07-4.71) |
| Chronic pancreatitis | 35 | 0 | 285 | 0.00 | - | - |
| 1 medical condition |  |  |  |  |  |  |
| No Pancreatitis | 2411 | 30 | 22410 | 1.34 | 1.00 | (reference) |
| Pancreatitis | 764 | 19 | 7052 | 2.69 | 2.61 | (1.28-5.30) |
| Acute pancreatitis | 747 | 19 | 6881 | 2.76 | 2.69 | (1.32-5.46) |
| Chronic pancreatitis | 17 | 0 | 171 | 0.00 | - | - |
| ≥2 medical conditions |  |  |  |  |  |  |
| No Pancreatitis | 1218 | 33 | 11258 | 2.93 | 1.00 | (reference) |
| Pancreatitis | 584 | 17 | 5255 | 3.24 | 1.09 | (0.46-2.58) |
| Acute pancreatitis | 565 | 17 | 5098 | 3.33 | 1.12 | (0.48-2.64) |
| Chronic pancreatitis | 19 | 0 | 158 | 0.00 | - | - |
| AMI, acute myocardial infarction; CI, confidence interval; HR, hazard ratio.  ^a^Adjusted for all covariates listed in Table 1.  ^b^Per 1000 person-years. | | | | | | |

| **Table S3.** The stratified analyses for the risk of stroke in patients with acute and chronic pancreatitis | | | | | | |
| --- | --- | --- | --- | --- | --- | --- |
|  | Long-term risk of stroke | | | | | |
|  | N | Events | Person-years | Incidence^b^ | HR | (95% CI)^a^ |
| Female |  |  |  |  |  |  |
| No Pancreatitis | 3284 | 190 | 28417 | 6.69 | 1.00 | (reference) |
| Pancreatitis | 808 | 70 | 6710 | 10.4 | 1.86 | (1.28-2.69) |
| Acute pancreatitis | 793 | 69 | 6585 | 10.5 | 1.88 | (1.30-2.71) |
| Chronic pancreatitis | 15 | 1 | 125 | 8.00 | - | - |
| Male |  |  |  |  |  |  |
| No Pancreatitis | 7541 | 359 | 66994 | 5.36 | 1.00 | (reference) |
| Pancreatitis | 1870 | 147 | 15795 | 9.31 | 1.83 | (1.45-2.31) |
| Acute pancreatitis | 1814 | 138 | 15355 | 8.99 | 1.74 | (1.37-2.21) |
| Chronic pancreatitis | 56 | 9 | 441 | 20.4 | 5.37 | (2.62-11.0) |
| Age 20-39 years |  |  |  |  |  |  |
| No Pancreatitis | 3119 | 25 | 29198 | 0.86 | 1.00 | (reference) |
| Pancreatitis | 776 | 24 | 7036 | 3.41 | 2.48 | (1.31-4.68) |
| Acute pancreatitis | 759 | 24 | 6868 | 3.49 | 2.53 | (1.34-4.77) |
| Chronic pancreatitis | 17 | 0 | 168 | 0.00 | - | - |
| Age 40-64 years |  |  |  |  |  |  |
| No Pancreatitis | 5036 | 216 | 45332 | 4.76 | 1.00 | (reference) |
| Pancreatitis | 1244 | 110 | 10581 | 10.4 | 1.83 | (1.45-2.33) |
| Acute pancreatitis | 1200 | 102 | 10253 | 9.95 | 1.75 | (1.37-2.24) |
| Chronic pancreatitis | 44 | 8 | 328 | 24.4 | 4.70 | (2.30-9.61) |
| Age ≥65 years |  |  |  |  |  |  |
| No Pancreatitis | 2670 | 308 | 20881 | 14.8 | 1.00 | (reference) |
| Pancreatitis | 658 | 83 | 4887 | 17.0 | 1.02 | (0.80-1.30) |
| Acute pancreatitis | 648 | 81 | 4819 | 16.8 | 1.00 | (0.78-1.29) |
| Chronic pancreatitis | 10 | 2 | 69 | 29.0 | 2.39 | (0.59-9.64) |
| 0 medical condition |  |  |  |  |  |  |
| No Pancreatitis | 7196 | 160 | 63452 | 2.52 | 1.00 | (reference) |
| Pancreatitis | 1330 | 32 | 10944 | 2.92 | 1.01 | (0.63-1.61) |
| Acute pancreatitis | 1295 | 30 | 10661 | 2.81 | 0.93 | (0.57-1.52) |
| Chronic pancreatitis | 35 | 2 | 283 | 7.07 | 4.03 | (0.99-16.4) |
| 1 medical condition |  |  |  |  |  |  |
| No Pancreatitis | 2411 | 201 | 21521 | 9.34 | 1.00 | (reference) |
| Pancreatitis | 764 | 72 | 6815 | 10.6 | 1.89 | (1.36-2.64) |
| Acute pancreatitis | 747 | 69 | 6662 | 10.4 | 1.86 | (1.33-2.60) |
| Chronic pancreatitis | 17 | 3 | 153 | 19.6 | 3.35 | (0.82-13.7) |
| ≥2 medical conditions |  |  |  |  |  |  |
| No Pancreatitis | 1218 | 188 | 10438 | 18.0 | 1.00 | (reference) |
| Pancreatitis | 584 | 113 | 4745 | 23.8 | 2.14 | (1.60-2.88) |
| Acute pancreatitis | 565 | 108 | 4616 | 23.4 | 2.09 | (1.56-2.82) |
| Chronic pancreatitis | 19 | 5 | 129 | 38.8 | 4.89 | (1.77-13.5) |
| CI, confidence interval; HR, hazard ratio.  ^a^Adjusted for all covariates listed in Table 1.  ^b^Per 1000 person-years. | | | | | | |
